# Analyzing Influencing Factors of Transfer Passenger Flow of Urban Rail Transit: A New Approach Based on Nested Logit Model Considering Transfer Choices

**DOI:** 10.3390/ijerph18168462

**Published:** 2021-08-10

**Authors:** Zhenjun Zhu, Jun Zeng, Xiaolin Gong, Yudong He, Shucheng Qiu

**Affiliations:** 1 College of Automobile and Traffic Engineering, Nanjing Forestry University, No.159 Longpan Road, Nanjing 210037, China; zhuzhenjun@njfu.edu.cn (Z.Z.); gongxiaolin_87@126.com (X.G.); heyudong@njfu.edu.cn (Y.H.); qsc@njfu.edu.cn (S.Q.); 2 School of Transportation, Southeast University, No.2 Dongnandaxue Road, Nanjing 211189, China

**Keywords:** transfer passenger flow, urban rail transit, transfer choice, nested logit model, Nanjing

## Abstract

With the continuous improvement of the operation line network of urban rail transit, analyzing influencing factors of transfer passenger flow of urban rail transit is critical to improve the transfer demand analysis of urban rail transit. Using data collected from questionnaires, transfer passenger flow surveys and smart cards, this study proposes an approach base on nested logit passenger flow assignment model considering transfer choice behaviours of passengers. The transfer passenger flow at seven transfer stations in Nanjing is obtained. Subsequently, this study investigates the potential influencing factors of transfer passenger flow, including the node degree, geographic location (located in the city center, urban fringe, suburbs or suburban fringe), economic location (distance from the city center) and transportation locations (if it is close to a transportation hub or in combination with the hub) of rail transit transfer stations. The results indicate that a positive correlation between the transfer passenger flow and the node degrees of transfer stations. However, the relationship between transfer passenger flow and the economic, geographic, and transportation locations of transfer stations is not clear. The finding have reference value for the network design of rail transit transfer stations and transfer facilities, and provide reference for the analysis of passenger flow under network operation.

## 1. Introduction

Urban rail transit is the most promising public transport mode for alleviating urban road traffic. Thirty-one Chinese cities have been operating 127 urban rail transit lines since 31 December 2016. The total operation mileage is currently 3934.8 km [1]. The urban rail transit system is in the stage of network operation in megacities in China including Beijing, Shanghai, Guangzhou and Shenzhen. However, the network operation is accompanied by an increase in the number of transfer and transfer passenger flow. The high transfer ratio demands better transfer passenger flow organization and a higher service level of transfer facilities and train operational schemes, especially during peak hours.

Over the past 20 years, automatic fare collection (AFC) has been extensively employed in urban rail transit around the world, especially in Europe and Asia. The application of AFC reduces operation costs and accelerates the speed of payment processing, and the generation of a large number of smart card data is beneficial for understanding passengers’ travel demands and characteristics. In the field of public transport, studies based on smart card data have focused on network planning [2,3], service evaluation [4,5] and the behaviour analysis of travellers [6,7]. These studies reveal that smart card data can accurately record the time and station and provide valuable information for our research.

Currently, the operation of urban rail transit commonly employs AFC systems to manage ticket data. According to passengers’ travel records collected by an AFC system, boarding and alighting of the trains by passengers and their respective time can be obtained, which provides explicit origin-destination (OD) information for passenger flow distribution. Urban rail transit in China primarily adopts an operation mode of integrated ticketing, and passenger travel intermediate information, such as transfer and route selection, cannot be calculated due to the difficulty of collection via AFC systems. Numerous studies of the calculation of passenger flow assignment with respect to urban rail transit have been conducted. For example, Poon et al. [8] proposed a dynamic passenger flow assignment model after considering the influence of congestion changes on the utility function during peak hours. Si et al. [9] considered influencing factors, including total travel time and transfer cost, in a logit-based passenger flow assignment model. Zhou and Xu [10] devised a passenger flow assignment model for urban rail transit based on the entry and exit time and the train operation constraints. In a study by Zhu et al. [11], a modified stochastic user-equilibrium assignment algorithm was proposed based on the framework of the method of successive averages. Kato et al. [12] employed the same preference data of urban rail route choice to compare the performance of six traffic assignment methods. In this study, travel time, transfer cost and congestion are included in the model, but the effect of fare on route choice is not considered.

Numerous studies on the relationship between urban rail transit ridership and influencing factors have been conducted [13,14,15]. In a study by Kuby [16], cross-sectional boarding data for 268 stations in nine US cities were collected and analyzed. The results revealed 10–12 significant factors that influence light-rail station boarding, such as employment, population, and park-and-ride spaces. Singhal et al. [17] utilized daily and hourly subway ridership data from New York City Transit and demonstrate the impact of weather on the variation in transit ridership. In studies by Sohn and Shim [18], Gutiérrez et al. [19] and Zhao et al. [20], direct prediction models were applied to analyze the influencing factors of passenger flow with Seoul, Madrid and Nanjing as case cities. By adopting a station-to-station analysis of Metro ridership, it was found by Choi et al. [21] that in the peak morning hours the population is a key variable for Metro boardings in an origin station, while employment becomes a key variable in the same origin station during evening peak hours. In a study by Chan and Miranda-Moreno [22], population density, average income, bus service connectivity, distance to central station, and service frequency are linked to trip production ridership during morning peak hours. Factors such as commercial and governmental land uses, bus connectivity, and transfer stations are associated with station attraction ridership during morning peak hours [23]. Lee et al. [24] analyzed the relationship between the ridership of the Seoul Metropolitan Subway and the land-use pattern of its station areas. The results show that the subway ridership at Seoul’s CBD and fringe areas is primarily influenced by density, whereas the sub-central area is generally affected by diversity. However, previous studies do not sufficiently address transfer passenger flow, and few studies have explored the influence of the network topology and location of transfer stations on transfer passenger flow.

To fill the important knowledge gaps, this study investigates the extraction of transfer passenger flow based on the transfer choice behaviours of passengers and the influencing factors for transfer passenger flow. The results of this study are valuable for the rail transit design of transfer stations and transferring facilities. The passenger flow assignment model, which is based on a nested logit model, is proposed after the generalized cost calculation and determination of effective routes. Seven rail transit transfer stations in the city of Nanjing, China have been analyzed. A survey of passengers was conducted to obtain information about their travel routes by rail transit at the seven transfer stations and to obtain data regarding the attributes of passengers, time on the subway train, perceived transfer time, number of transfers, longest acceptable travel time for this trip and familiarity with the rail transit network. The third section discusses the relationship between transfer passenger flow and the characteristics of the network topology and location of transfer stations. The final section presents conclusions regarding the main findings and proposes future research targets.

## 2. Data Collection

**Figure 1:**
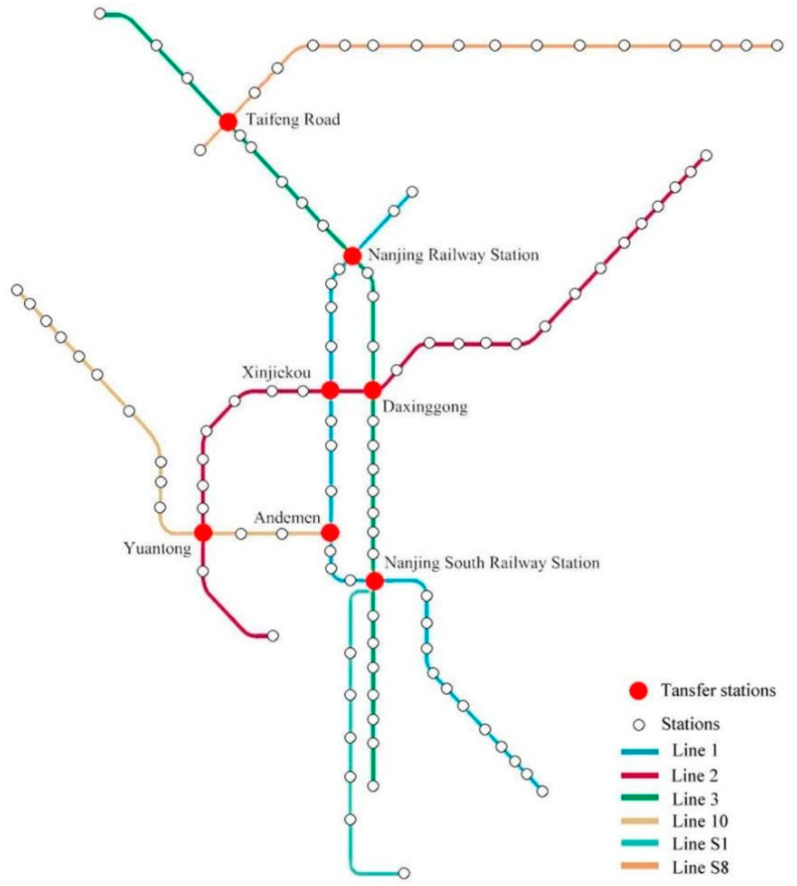
Nanjing rail transit network (2016). The figure displays the network topology and transfer relationships between different rail transit lines (based on the Nanjing subway rail route map).

Nanjing is selected as an example to analyse influencing factors of transfer ridership of urban rail transit. Nanjing is located in the Yangtze River Delta Region, and it is the second largest city in Eastern China, next to Shanghai. Six urban rail transit lines are currently in operation with 121 stations, of which seven stations are transfer stations, as shown in Figure 1, which shows the operation status of Nanjing Metro Company, Nanjing, China. After performing this investigation, this study observed substantial differences in the transfer passenger flow of these transfer stations. This study focuses on the cause of this difference, proposes the hypothesis that characteristics of the network topology and the locations of the transfer stations may influence transfer passenger flow.

To understand the influencing factors of passengers’ route choices, a questionnaire survey of passengers about their travel routes by rail transit was conducted at seven of the most important interchange transfer stations in Nanjing, China in June 2016, which covered their personal characteristics, travel characteristics, interchange characteristics and scenario preferences. A total of 312 valid questionnaires were collected from 350 respondents. The following data were collected:

(a) Personal characteristics: gender (male, female), age (18 or younger, 19–30, 31–55, 56 and over), and occupation (student, public servant, service industry, commerce, industry, unemployed/retired, others).

(b) Trip characteristics: boarding station, alighting station, time on the subway train and familiarity with rail transit network.

(c) Transfer characteristics: perceived transfer time, number of transfers, transfer walking time, and waiting time.

(d) Scenario preferences: longest acceptable travel time for this trip and transfer influencing factors.

The data were analyzed anonymously; therefore, the authors had no access to personal identifying information. The smart card data for April 2015 were provided by Nanjing Metro Group Co., Ltd. This study investigated the transfer passenger flow of seven stations during peak hours in June 2016.

## 3. Methodology

### 3.1. Transfer Ridership Extraction

**Figure 2:**
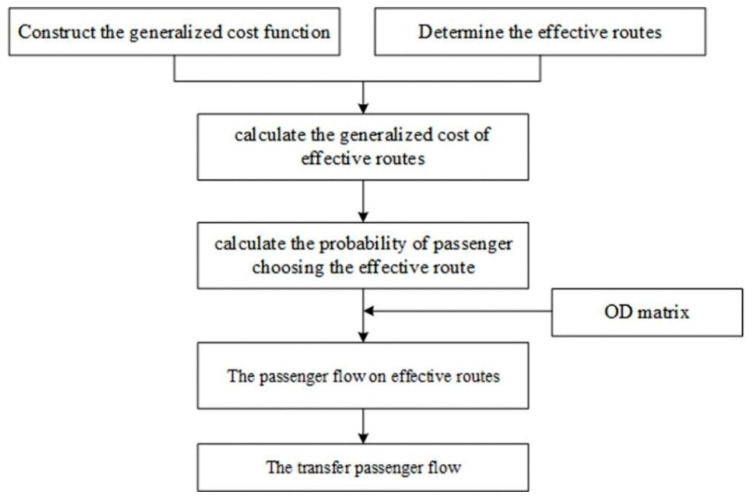
The flow chart of nested logit passenger flow assignment model.

The nested logit ridership assignment model was used to obtain the transfer passenger flow for the transfer stations; specific steps are shown in Figure 2. On the basis of the survey, the parameters of the cost function (fare, number of transfers, etc.) and the effective routes in the trip are obtained. Based on the selection of effective routes and travel costs, combined with the OD matrix, the potential passenger flow data for each route is obtained. After that, the changing passenger flow is obtained.

### 3.2. Generalized Cost Function

Passenger route selection is influenced by many factors. According to the survey, travel time, fares, and the number of transfers are the main factors.

#### 3.2.1. Travel Time

Travel time is the total travel time from the departure station to the destination station, including operating time, dwell time at intermediate stations, transfer walking time and transfer waiting time. Therefore, passenger travel time (in minutes) by rail transit can be expressed as Equation (1):

(1) $$T_{\mathrm{ij}}=\sum_{}^{N+1}t_{ab}^{l}+\sum S_{k}^{}+\sum_{}^{N}t_{walk}^{l,m}+\sum_{}^{N}t_{wait}^{l,m}$$

where *i* and *j* represent the departure station and destination station, respectively; $t_{ab}^{l}$ represents the operation time between station *a* and station *b* in line *l*; *a* is the boarding station; b is the disembarking station;N is the number of transfers; $S_{k}$ is the dwell time at each intermediate station; $S_{k}$ is the transfer walking time from line *l* to line *m*; and $t_{wait}^{l,m}$ is the average waiting time when passengers transfer from line *l* to line *m*.

The transfer waiting time is related to train operation planning [25]. To simplify calculations, half of the departure interval is taken as the transfer passenger waiting time, as shown in Equation (2):

(2) $$t_{wait}^{l,m}=0.5\cdot f_{m}$$

where $f_{m}$ represents the train’s average departure interval of line *m*.

#### 3.2.2. Ticketing System and Fares

In an urban rail transit system, a ticketing system and fares primarily refer to integrated ticketing or segmented pricing for one complete trip. If segmented pricing is employed, then the fare level for different mileages will influence passengers’ travel route selection. Nanjing adopts segmented pricing, the unit of valuation is Yuan.

#### 3.2.3. Number of Transfers

A lower number of transfers results in a greater chance of routes being selected. Passengers have different levels of sensitivity regarding the transfer time and the number of transfers; as the number of transfers increases, passengers’ perception of costs successively increase. This study assumes that the transfer time should be increased for a transfer penalty if only one transfer is required. If two transfers are required, then the second transfer penalty is greater than the first transfer penalty, and the third transfer penalty is greater than the second transfer penalty, which are successively analysed. Therefore, travel time with the transfer penalty condition can be expressed as Equation (3):

(3) $$T_{\mathrm{ij}}{}^{'}=\sum_{}^{N+1}t_{ab}^{l}+\sum S_{k}^{}+\sum_{}^{N}{\left(n_{p,r}\right)}^{\beta -1}(t_{walk}^{l,m}+t_{wait}^{l,m})$$

where $r_{p,r}$ is the cumulative number of transfers at the transfer station p of route r between the OD, and β is the parameter to be calibrated, which can be obtained via surveys; it is a penalty factor for the number of passenger transfers to reflect the increase in cost of the transfer, so the range of values is set from 1 to 2.

To ensure that fares and travel time have the same contribution rate as the total cost, the travel time is converted into a value with the same order as the fares. The generalized cost function of passengers’ travel routes is proposed in Equation (4):

(4) $$C_{r}^{ij}=T_{\mathrm{ij}}{}^{'}/10+F$$

where $C_{r}^{ij}$ is the total cost of route *r* between OD and F is the one-way ticket fare. where 10 is used to perform a preliminary processing of the time so that it can be normalized.

#### 3.2.4. Determination of Effective Routes

Passengers can select multiple connecting routes between their OD to travel when urban rail transit lines develop into a network. Due to the significant differences among the travel time, transfer time and number of transfers along each route, the travel time and the number of transfers along some routes exceed the range that is acceptable to passengers. Therefore, passengers will only select some routes as their options; these routes are effective routes. This paper adopts traversal algorithms, which are based on graphs and provide corresponding improvements; they are suitable for searching for the effective routes of a rail transit network based on the number of transfers.

In addition to determining whether a loop exists among the routes to the destination, routes that contain loops are excluded when using traversal algorithms to search for effective routes. The threshold of effective routes should also be determined. A criticality is set based on the cost of the shortest route. When the absolute or comparative difference between the route cost and the shortest route cost exceeds the threshold, the route is excluded, and the remaining routes are regarded as effective routes that should satisfy Equations (5) and (6):

(5) $$C_{k}^{ij}\leq (1+f_{\max}^{\left(1\right)})\cdot C_{\min}^{ij},f_{\max}^{\left(1\right)}>0$$

(6) $$C_{k}^{ij}\leq C_{\min}^{ij}+f_{\max}^{\left(2\right)},f_{\max}^{\left(2\right)}>0$$

where $f_{\max}^{\left(1\right)}$ is a comparative threshold, $f_{\max}^{\left(2\right)}$ is an absolute threshold, $C_{\min}^{ij}$ is the shortest route cost between the OD pairs *ij* and $V^{\left(k\right)}$ is the cost of route *k* between the OD pairs *ij*.

According to the characteristics of urban rail transit, this paper proposes traversal algorithms based on breadth-first search algorithms to search for effective routes. $V^{\left(k\right)}$ is defined as the storage critical node of the *k*th layer that is searched and satisfies the constraints of the effective routes. $V_{i}{}^{\left(k\right)}$ is the *i*th critical node of the *k*th layer; n(k) is the number of critical nodes at the *k*th layer. $V_{ij}{}^{\left(k\right)}$ is the *j*th subsequent node of the *i*th critical node at the *k*th layer. $V^{T}$ is a set of critical nodes, and *m* is the number of transfer stations. The calculation steps of the algorithms are as follows:

Step 1: Use the shortest route algorithm to calculate the shortest route cost $C_{\min}^{ij}$ between OD, and set a threshold value for $f_{\max}^{\left(1\right)}$ and $\;S=\left\{\left(A_{1}{}^{}\times B_{1}\right),\;\left(A_{1}\times B_{2}\right),\;\ldots ,\;\left(A_{3}\times B_{r}{}_{-1}\right),\;\left(A_{3}\times B_{r}\right)\right\}$. Initialize and let k=0, i=j=1, $V^{\left(k\right)}=\left\{V_{i}{}^{\left(k\right)}\right\}$, $V^{T}\neq$Ø, and $V_{1}{}^{\left(0\right)}=r$.

Step 2: Traverse *V_ij_*^(*k*)^ and calculate the corresponding cost $C\left(r,V_{ij}{}^{\left(k\right)}\right)$, and then go to Step 3; If $V_{i}{}^{\left(k\right)}$ has been traversed, go to Step 6.

Step 3: Determine whether the route is effective. If it is effective, then go to Step 4; otherwise, abandon the branch, let *i* = *i* + 1, and go to Step 2.

Step 4: Determine the node attributes of $V_{ij}{}^{\left(k\right)}$. If $V_{ij}{}^{\left(k\right)}$ is a termination node, then record the route, calculate the corresponding cost, let *j*= *j* + 1, and go to step 2. If $V_{ij}{}^{\left(k\right)}$ is an ordinary node, then let j=j+1, and go to step 2. If $V_{ij}{}^{\left(k\right)}$ is a transfer node, $V^{T}=V^{T}\cup V_{ij}{}^{\left(k\right)}$, let j=j+1 and m=m+1, and go to step 2.

Step 5: If $V_{ij}{}^{\left(k\right)}$ has been traversed, then let i=i+1 and j=1, and go to step 2.

Step 6: Let  k=k+1, i = 1, j = 1, and $V^{\left(k\right)}=V^{T}$. If $V^{\left(k\right)}\neq$*Ø*, then go to step 2; otherwise, the algorithm terminates.

#### 3.2.5. Route Choice Model

The nested logit model is a modified form of the multinomial logit model, which sets up a virtual layer to classify factors with similar properties as a class, places them in the same layer, and abstracts them into a tree structure diagram to overcome the IIA characteristic of the multinomial logit model.

**Figure 3:**
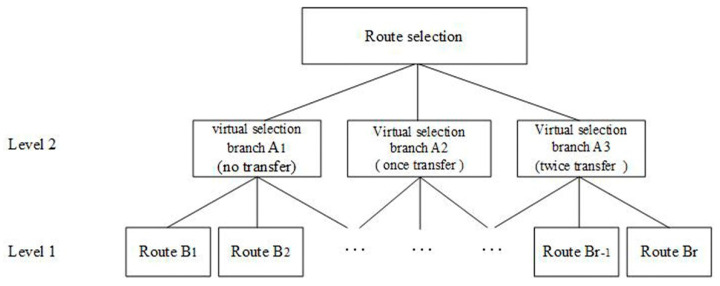
Structure of the double-nested logit model.

This study assumes that passengers will choose the number of transfers within a route and choose the specific route scheme for the number of transfers. In the early operation stages of a rail transit network, such as that in Nanjing, the maximum number of transfers that passengers can accept is two transfers. Therefore, this study constructs the upper virtual branches of the nested logit model for the urban rail transit route selection as consisting of no transfers, one transfer and two transfers, as shown in Figure 3.

Assume that the route selection scheme *S* is a set of selections composed of Level 1 and Level 2, which can be expressed as *S = {(A_1_ × B_1_), (A_1_ × B_2_), …, (A_3_ × B_r−1_), (A_3_ × B_r_)}*. Based on the theory of stochastic utility, every passenger will choose branches of the highest utility. The stochastic utility $U_{k}$ can be expressed as Equation (7):

(7) $$U_{k}=V_{k}+\varepsilon_{k},\;k\in S$$

where $V_{k}$ is the effectiveness determined by the passengers at the *k*th route, and $\varepsilon_{k}$ is the stochastic error term.

Assume that the utility of a route scheme is $U(A_{p},B_{q})$, as shown in Equation (8), and the utility of the virtual selection branches $A_{p}$ is $U(A_{p})$. Under $A_{p}$ of Level 2, the utility is $U(A_{p},B_{q})$ when choosing $B_{q}$ at the lower level.

(8) $$U(A_{p},B_{q})=U(A_{p})+U(B_{q}|A_{p})$$

Thus, based on the theory of stochastic utility,$U(A_{p},B_{q})$ is expressed as Equation (9):

(9) $$\begin{array}{l} U(A_{p},B_{q})=V(A_{p})+V(B_{q}|A_{p})+\varepsilon (A_{p})+\varepsilon (B_{q}|A_{p})\\ V(A_{p})=\sum_{h=1}^{H}\theta_{h}x_{ph}\\ V(B_{q}|A_{p})=\sum_{l=1}^{L}\beta_{l}x_{ql} \end{array}$$

where $V(A_{p})$ represents the determined items of the utility value when choosing $A_{p}$; $V(B_{q}|A_{p})$ represents the determined items when choosing $B_{q}$ at the lower level and $A_{p}$ at the upper level; $\varepsilon (A_{p})$ and $\varepsilon (B_{q}|A_{p})$ are the corresponding random items, which are independent of each other and subjected to the Gumbel distribution. *H* and *L* are the number of the upper characteristic vector and lower characteristic vector, respectively; $\theta_{h}$ and $\beta_{l}$ are the unknown upper parameter vector and the lower characteristic vector, respectively; and $x_{ph}$ and $x_{ql}$ are the upper characteristic vector and the lower characteristic vector, respectively, which are factor values of the model determined via the chi-square test or the Student’s *t*-test.

According to probability theory, the probability that $A_{p}$ and $B_{q}$ are simultaneously selected can be expressed as Equation (10):

(10) $$P(A_{p},B_{q})=P(A_{p})\cdot P(B_{q}|A_{p})$$

where $P(A_{p})$ is the probability of $A_{p}$, and $P(B_{q}|A_{p})$ is the probability of choosing *B_q_* at the lower level under the condition of *A_p_*, which has been chosen at the upper level.

When $\varepsilon (A_{p})$ and $\varepsilon (B_{q}|A_{p})$ are subjected to double exponential distribution, whose mean values are 0, the variances are $\delta_{2}^{2}$ and $\delta_{1}^{2}$, respectively. $P(A_{p},B_{q})$ can be expressed as Equation (11):

(11) $$P(A_{p},B_{q})=\frac{\exp\left\{\lambda_{2}[(V(A_{p})+V^{*}(A_{p})]\right\}}{\sum_{p=1}^{3}\exp\left\{\lambda_{2}[(V(A_{p})+V^{*}(A_{p})]\right\}}\cdot \frac{\exp[\lambda_{1}V(B_{q}|A_{p})]}{\sum_{q=1}^{r}\exp[\lambda_{1}V(B_{q}|A_{p})]}$$

where $\lambda_{1}$ is only related to the probability item variance of the lower utility $\delta_{1}^{2}$, $\lambda_{1}^{2}=\pi^{2}/6\delta_{1}^{2}$; $\lambda_{2}$ is related to the probability item variance of the upper utilities and lower utilities, $\lambda_{2}{}^{2}=\pi^{2}/6(\delta_{1}^{2}+\delta_{2}^{2})$; $U^{*}(A_{P})=\max(V(B_{q}|A_{p})+\varepsilon (B_{q}|A_{p}))$ is the synthetic utility, which is subjected to the double exponential distribution, whose mean value is $V^{*}(A_{p})=\frac{1}{\lambda_{1}}\ln\sum_{q=1}^{r}\exp(\lambda_{1}V(B_{q}|A_{p}))$, and the variance is $\delta_{1}^{2}$.

According to the constructed selection tree, the upper characteristic variables of the nested logit model are the transfer time and number of transfers, whereas the lower characteristic variables are the travel time and fares. The upper cost, lower cost and generalized cost of the nested logit model can be, respectively, expressed as follows:

(12) $$C2_{k}=\frac{1}{10}\sum_{}^{N}{\left(n_{p,r}\right)}^{\beta -1}(t_{walk}^{l,m}+t_{wait}^{l,m})$$

(13) $$C1_{k}=\frac{1}{10}(\sum_{}^{N+1}t_{ab}^{l}+\sum S_{k})+F$$

(14) $$C_{k}=C1_{k}+C2_{k}$$

Since the cost has a negative correlation with effectiveness, Equation (11) can be rewritten as Equation (15):

(15) $$P=\frac{\exp[-\lambda_{2}(C2_{k}+C_{h}^{*})]}{\sum_{h\in H}\exp[-\lambda_{2}(C2_{g}+C_{h}^{*})]}\cdot \frac{\exp(-\lambda_{1}C1_{k})}{\sum_{g\in R}\exp(-\lambda_{1}C1_{g})}$$

where R is the set of effective routes between OD, and $C_{h}^{*}=-\frac{1}{\lambda_{1}}\ln\sum_{g\in R}\exp(-\lambda_{1}C1_{g})$.

By the statistical analysis of the survey results to determine the model parameters, the values of the parameters are obtained, β = 1.4, $f_{\max}^{\left(1\right)}$ = 0.3, $f_{\max}^{\left(2\right)}$ = 2, $\lambda_{1}$ = 1, and $\lambda_{2}$ = 0.5.

### 3.3. Influencing Factors of Transfer Passenger Flow

Factors affecting transit passenger flow were classified into four types in the previous study [12,14,16]: (1) land use, such as population, employment, and the floor area of buildings; (2) external connectivity, for example, road density and the distance from a station to the city center; (3) intermodal connection, such as the number of bicycle P&R spaces; and (4) station context, for instance, “terminal or not” and “transfer or not”.

The research object of this study is to determine the factors that affect the transfer passenger flow of rail transit, where the transfer passenger flow refers to the sum of the transfer passenger flow between different lines within the rail transit transfer station, regardless of the transfer between rail transit and other external modes of transportation. Consequently, there is no obvious correlation between transfer passenger flow and land use around the transfer station, external connectivity (except for the distance from the city center, which will be classified as location factors below), intermodal connection, and station context.

When rail transit passengers travel from one line to another line, they choose where to transfer according to the line connection and the distribution of transfer stations, namely, the network topology. Location elements of transfer stations are likely to be related to transfer passenger flow.

#### 3.3.1. Characteristics of the Network Topology

**Figure 4:**
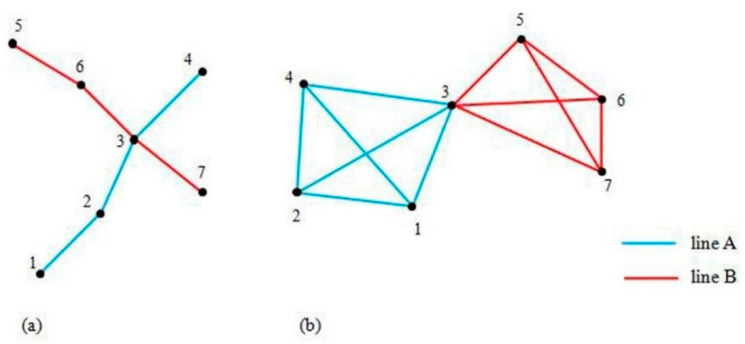
(**a**) Explanation of space *L*; (**b**) explanation of space *P*.

To analyze the characteristics of a rail transit network, a proper network topology should be defined. A network topology model primarily uses the space *L* and the space *P* in complex network studies [26,27]. An explanation of space *L* and space *P* is shown in Figure 4. Space *L* consists of nodes that represent bus or rail transit stations, and a link between two nodes exists if they are consecutive stations on a route. The node degree in this topology is the number of directions that a person can take from a given node. Although the nodes in space *P* are the same as the nodes in space *L*, an edge between two nodes indicates that a direct bus or rail transit route links them. Therefore, the node degree of the station *i* in this topology is the total number of nodes that are reachable using a single route without a transfer. Space *P* abstracts stations into nodes if no transfer exists between two stations in the traffic network and connects the two stations, and stations of different lines are classified by taking the transfer station as the centre. Stations on the same line are clustered into one group, and similar nodes are connected. Stations on different lines are classified, and the shortest route among nodes of different types is larger than 1, which is convenient and intuitive to study the accessibility between stations and the minimum number of transfers. The physical meaning of the characteristic parameters of space *P* is relatively explicit and primarily reflects the transfer characteristics of an urban rail transit network.

The node degree is the simplest and most important concept that depicts the properties of the node. A larger node degree signifies a greater importance in the network. The node degree of space *P* in the Nanjing rail transit network can be calculated, as shown in Table 1.

#### 3.3.2. Location Elements of Transfer Stations

**Figure 5:**
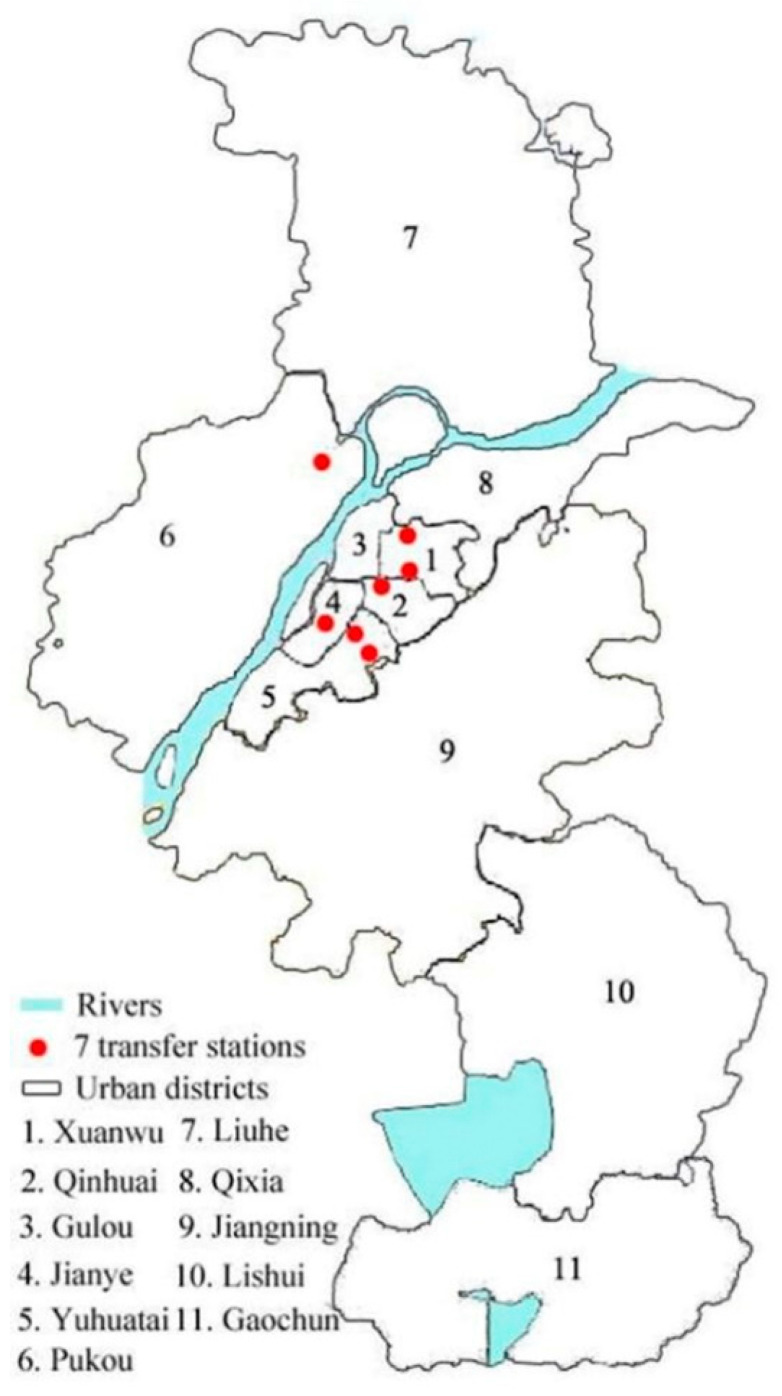
Location of rail transit transfer stations in Nanjing.

Location is a compound concept that includes position, transportation, economy, and different location factors; the combinations of these factors form different location conditions. The geographic location (located in the city centre, urban fringe, suburbs or suburban fringe), economic location (distance from the city centre) and transportation location (close to a transportation hub or in combination with it) of rail transit transfer stations in a city influence transfer passenger flow and generate differences in passenger flow intensity among transfer stations and locations, as shown in Figure 5.

## 4. Results and Discussions

### 4.1. Transfer Ridership and Node Degree

In the rail transit network, Nanjing South Railway Station has the maximum node degree; Nanjing Railway Station, Daxinggong and Xinjiekou have the second largest node degree; Taifeng Road, Yuantong and Andemen have the third largest node degree; and Yuantong has the lowest node degree.

**Figure 6:**
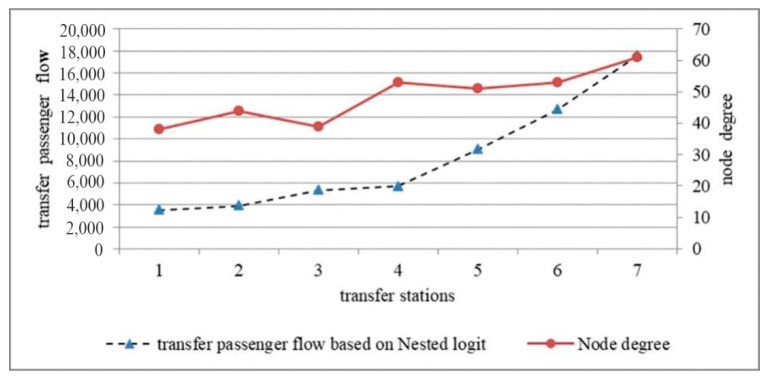
The relationship between transfer passenger flow and node degree.

A positive correlation between the transfer passenger flow and the node degree of transfer stations is observed, as shown in Figure 6; however, the following special cases are noted:

In Figure 6, 1–7 refer to Yuantong, Taifeng Road, Andemen, Daxinggong, Xinjiekou, Nanjing Railway Station, and Nanjing South Railway Station, respectively.

(1) Nanjing Railway Station, Daxinggong and Xinjiekou have similar node degrees, and Daxinggong is closer to the city centre than Nanjing Railway Station. However, the transfer passenger flow of Nanjing Railway Station is significantly larger than that of Daxinggong and Xinjiekou. As Nanjing Railway Station is a large-scale transportation hub, it has a better transportation location than Daxinggong and Xinjiekou. Nanjing Railway Station is the gateway into urban districts by urban rail transit for passengers in the northern suburbs of Nanjing. Passengers who live in the northern suburbs and work in the urban districts must take line 3 and transfer to line 1 at the Nanjing Railway Station. In the morning rush hour, therefore, the number of commuter passengers is especially high in the total transfer passenger flow. When we investigated the transfer passenger flow at Nanjing Railway Station, it was discovered that the number of passengers who transferred from line 3 to line 1 was extremely large during the morning rush hour and substantially outweighed the number of passengers who transferred from line 1 to line 3. This finding may be attributed to the fact that only two stations of line 1 are located north of Nanjing Railway Station.

(2) The node degree of Xinjiekou is slightly smaller than the node degree of Daxinggong, whereas the transfer passenger flow of Xinjiekou is higher than the transfer passenger flow of Daxinggong, as the former has a better economic location than the latter.

(3) The node degree of Andemen is smaller than the node degree of Taifeng Road, as the transfer passenger flow of Andemen is higher than the transfer passenger flow of Taifeng Road. As Taifeng Road is located in suburbs, its passenger flow is relatively small. In addition, Andemen is located in the urban fringe and is closer to the city centre than Taifeng Road; thus, the former has a better economic location and geographic location than the latter.

### 4.2. Transfer Ridership and Location Elements

The city centre of Nanjing is located in the Xinjiekou area. Taking Xinjiekou station as the reference point, the distances to the rail transit line from other transfer stations are measured as their economic locations. Among them, Taifeng Road is located in Xinjiekou, which is 17 km north of the city centre, and is the furthest from the city centre. Daxinggong is 1.02 km from Xinjiekou in the east and is closest to Xinjiekou. Nanjing South Railway Station is located in Xinjiekou, 11.71 km south of the city center. The distance to Yuantong from Xinjiekou is less than the distance to Nanjing South Railway Station. The Nanjing Railway Station and Andemen are located approximately the same distance from Xinjiekou.

Regarding geographic location, Xinjiekou and Daxinggong are located in the city centre district. Nanjing Railway Station, Nanjing South Railway Station, Andemen and Yuantong are located in the urban fringe, and Taifeng Road is located in the suburbs. For a quantitative analysis, assume that the geographic location of Xinjiekou and Daxinggong is 1, the geographic location of Nanjing Railway Station, Nanjing South Railway Station, Andemen and Yuantong is 2, and the geographic location of Taifeng Road is 3.

Nanjing Railway Station and Nanjing South Railway Station comprise a combination of a rail transit station and a large-scale transportation hub. For a quantitative analysis, assume that their transportation location is 1, and the transportation location of other transfer stations is 0.

**Figure 7:**
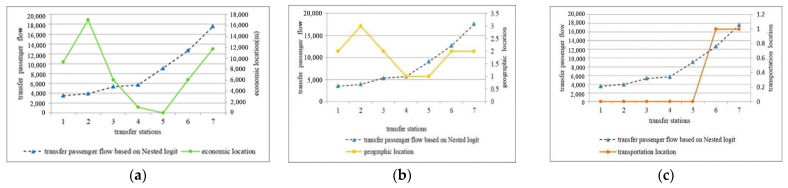
(**a**) The relationship between transfer passenger flow and economic location; (**b**) the relationship between transfer passenger flow and geographic location; (**c**) the relationship between transfer passenger flow and transportation location. In the figure, 1–7 refer to Yuantong, Taifeng Road, Andemen, Daxinggong, Xinjiekou, Nanjing Railway Station, and Nanjing South Railway Station, respectively.

No distinct relationship between transfer passenger flow and the economic location, geographic location, transportation location of transfer stations is observed, as shown in Figure 7a–c.

## 5. Conclusions

This study discussed the factors that affect the transfer passenger flow at the station level in Nanjing, China, which is in the early network operation phase. The characteristics of the network topology and the location of transfer stations may influence the transfer passenger flow [28,29]. We verified this hypothesis using data from several sources, a questionnaire survey and a transfer passenger flow survey conducted at the seven transfer stations in Nanjing as well as smart card data for April 2015 provided by the Nanjing Metro Group Co., Ltd. The correlation analysis and comparative analysis approach were adopted to explore the relationship between transfer passenger flow and variables that measure characteristics of the network topology and location of the transfer stations. The legitimacy of the results was discussed based on our insights about Nanjing.

The study concluded that a positive correlation existed between the transfer passenger flow and the node degree of transfer stations, whereas no obvious relationship existed between transfer passenger flow and economic location, geographic location, and transportation location of the transfer stations. Our findings have some implications. Characteristics of the network topology, such as node degree, should be investigated to improve the transfer demand analysis of urban rail transit. For station designers, transfer stations with high node degrees should have transfer facilities with a greater capacity inside the station.

However, this study has several limitations. First, congestion in the subway train influenced the route choice of passenger travel; however, the congestion effect is omitted in the generalized cost calculation. To simplify the calculation process, this study only considered the frequency of train departures and did not take into account train scheduling. Second, many other factors that influence transfer passenger flow are not included due to the lack of data, such as accessibility, land use, employment and population density and network forms of urban rail transit. Third, simplified analysis of Nanjing subway network is performed in this study using nest logit. However, when the structure of the urban rail transit network is more complex, passenger transfer choices and passenger flow distribution in the network show dynamic changes. The hyperpath and dynamic passenger flow distribution are typical concepts that consider the dynamic changes and relative differences between rail lines and stations. Introducing these concepts to future researches could improve the adaptability of this study.

## Figures and Tables

**Table 1:** Node degree of space *P* in the Nanjing rail transit network.

Name of Stations	Node Degree
Taifeng Road	44
Nanjing Railway Station	53
Xinjiekou	51
Daxinggong	53
Yuantong	38
Andemen	39
Nanjing South Railway Station	61

## Data Availability

The data presented in this study are available on request from the corresponding author.
